# Molecular Mechanisms and Clinical Implications of Noncoding RNAs in Cancer

**DOI:** 10.3390/ncrna10040037

**Published:** 2024-06-25

**Authors:** Jin Wang, Xiaomeng He, Christopher Corpe

**Affiliations:** 1 Central Laboratory, Zhongshan Hospital (Xiamen), Fudan University, Xiamen 361015, China; 2 Shanghai Public Health Clinical Center, Fudan University, 2901 Caolang Road, Jinshan District, Shanghai 201508, China; 3 Department of Nutritional Sciences, King’s College London, 150 Stamford Street, Waterloo, London SE1 9NH, UK

Noncoding RNAs (ncRNAs), which include small nuclear RNAs (snRNAs), small nucleolar RNAs (snoRNAs), microRNAs (miRNAs), long noncoding RNAs (lncRNAs), and circular RNAs (circRNAs), are RNA molecules that arise from genomic regions without protein-coding potential and display a variety of mechanisms and functions by regulating gene expression at the transcriptional, RNA processing, and translational levels and participating in virtually all cellular processes [1,2]. Dysregulated ncRNAs are involved in many complex human diseases and exert distinct functions, such as oncogenic and tumor-suppressive roles [1,3,4,5]. In this Special Issue, we gathered six articles—three original research contributions, one communication, and two review articles—that focused on the latest findings and important perspectives of the molecular mechanisms and clinical implications of ncRNAs in various cancers, including the discovery and structural prediction of novel miRNAs, as well as the mechanism of miRNAs and lncRNAs in cancer proliferation, metastasis, invasion, and drug resistance and their clinical application as potential new biomarkers and therapeutic drugs. First, Sajeev et al. [6] provided a fascinating review of the roles of ncRNAs, including miRNAs, lncRNAs, and circRNAs, in the occurrence, progression, and metastasis of head and neck cancer (HNC), with a focus on the crosstalk between miRNAs/lncRNAs and Wnt/β-catenin signal transduction, which regulates cell proliferation, EMT, invasion, migration, chemical resistance, and radiation resistance in HNC. Sajeev et al. [6] also highlighted the importanecet of understanding the role of nCRNAs in designing breakthrough therapeutic interventions for this malignant tumor.

miRNAs are short-stranded RNA molecules with a length of approximately 22 bp and are highly conserved across species; they are also associated with epigenetic silencing and can usually bind to target mRNAs to trigger translation inhibition or induce mRNA degradation [7]. Currently, approximately 2800 human miRNAs have been annotated in public repositories [8]. With the development of high-throughput technology, an increasing number of novel miRNAs with increased tissue specificity and decreased expression levels have been discovered [9,10,11]. The abnormal expression of miRNAs is closely related to the occurrence and development of various diseases, including cancer [12], and exosomal miRNAs can serve as potential biomarkers for cancer diagnosis and prognosis [13]. In the present research on miRNAs, Minutentag et al. [14] analyzed small RNA sequence data and the miRMaster algorithm as well as independent datasets to analyze and validate 15 novel miRNAs with potential biological relevance in colorectal cancer (CRC). The authors evaluated the expression patterns of these genes based on tumor location, prognosis, and putative target genes. Among them, four novel miRNAs (miR-13844-5p, miR-590-5p, miR-7154-5p, and miR-5035-3p) were differentially expressed between distal and proximal CRC. miR-13172-3p and miR-3345-5p were only expressed in colorectal tissues and exhibited tissue specificity. The associations between seven novel miRNAs (miR-13844-5p, miR-590-5p, miR-3345-5p, miR-13172-3p, miR-7154-5p, miR-766-3p, and miR-8861-5p) and 81 targets were also revealed as potential biomarkers for CRC. Jonas [15] also conducted a cytological functional verification of miR-4646-5p via three-dimensional breast cancer spheroid expression screening for the first time. miR-4646-5p mainly acts as a tumor suppressor in triple-negative breast cancer (TNBC) and targets the cholesterol transporter GRAMD1B to regulate the growth, proliferation, and migration of TNBC cells and the formation of endothelial cells in vitro and to induce apoptosis. Multiple other direct and indirect targets that exert miR-4646-5p tumor-suppressive effects, such as the cytokines and chemokines GCSF and IL6, which have tumor-promoting functions, were also explored, providing a theoretical basis for the combined treatment of TNBC with miR-4646-5p and immunotherapy, such as checkpoint inhibitors. PremiR-675 is a miRNA expressed by H19 lncRNA, and its abnormal expression is associated with various diseases, such as cancer and cardiovascular and neurological disorders [16]. Furthermore, Dey’s research revealed that premiR-675 could fold into a typical stem–loop helical conformation and has binding sites for FUS, SRSF1, SRSF9, FXR2, LIN28B, and HUR proteins, which can be further used for structure-based drug design. This is the first study to clarify the molecular mechanism by which H19 lncRNA promotes the activity of premiR-675 by modifying the conformation of premiR-675, which will further help to understand the biological mechanism of premiR-675 in detail.

lncRNAs, also known as competitive endogenous RNAs (ceRNAs), are RNA molecules with lengths greater than 200 bp that lack coding sequences and are poorly conserved across species [17]. Compared with miRNAs, lncRNAs use multiple mechanisms to regulate gene expression, including recruiting RNA polymerase II and various transcription factors, regulating mRNA decay, or directly binding to miRNAs to exert a “sponge effect” [18]. The interactions of lncRNAs and miRNAs in gene expression and regulation are shown in Figure 1. A large number of studies have shown that lncRNAs are dysregulated in various types of cancer, mainly by regulating signaling cascades at the transcriptional and translational levels to participate in biological processes such as tumorigenesis, progression, metastasis, and drug resistance [4,19,20]. Dysregulated lncRNAs in tumors also play a crucial role in immune regulation and the tumor microenvironment of cancer [21,22,23,24]. Also inthis Special Issue for lncRNAs, Peña-Flores et al. [18] reviewed the most recent comprehensive information on the molecular behavior of long intergenic noncoding RNA reprogramming regulators (Linc-ROR); summarizing the functions and regulatory mechanisms of Linc-ROR in different types of cancer; analyzing and discussing the status of Linc-ROR in human cancer proliferation, EMT, invasion, metastasis, and drug resistance; exploring the clinical relevance of Linc-ROR in various cancer types, including clinical staging, tumor metastasis, lymph node metastasis, and vascular invasion; and clarifying the potential use of Linc-ROR as a cancer biomarker. Chemotherapy resistance is a common phenomenon in cancer treatment, and lncRNAs play an important role in regulating cellular sensitivity to drug chemotherapy [25]. Finally, Azwar et al. [26] applied cDNA microarray technology and bioinformatics analysis to identify differentially expressed lncRNAs and mRNAs in 5-fluorouracil (5-FU)-resistant SW480/DR cells and 5-FU-sensitive SW480/DS cells. In their study they screened and identified potential lncRNAs involved in the sensitivity of colon cancer cells to 5-FU chemotherapy drugs, which may participate in chemical resistance by regulating exocytosis. This is the first study to show that the lncRNAs GNAS-AS1 and MIR205HG participate in regulating colon cancer cell sensitivity to 5-FU chemotherapy by promoting the release of exosomes into the intercellular matrix. This information is beneficial for clarifying the mechanism by which exosomes affect colon cancer cell resistance to 5-FU chemotherapy.

In summary, as a diverse group of RNA products, ncRNAs play important roles in the occurrence of various diseases. A deeper understanding of their specific mechanisms is crucial for the development of refined treatment strategies. This Special Issue not only provides information about the current research status of new functional miRNAs and lncRNAs in cancer, but also provides insight into the future directions of research on miRNAs and lncRNAs. Moreover, these studies reveal the applicability of miRNAs and lncRNAs as prognostic indicators, biomarkers, and therapeutic targets for various cancers. We look forward to obtaining new applications and useful data in this constantly developing field.

## Figures and Tables

**Figure 1:**
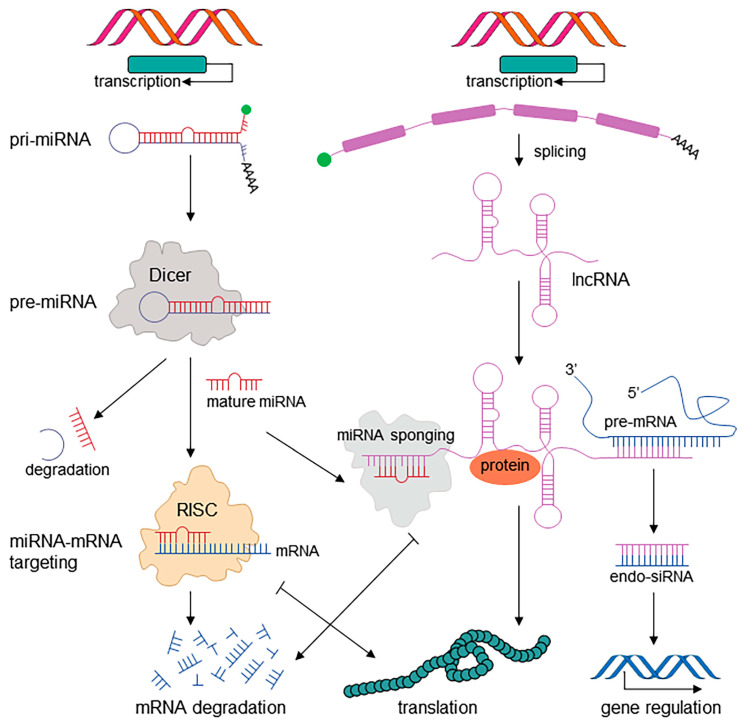
The interaction of miRNAs and lncRNAs in gene expression and regulation.
